# A comparative study to evaluate microstrain of low-profile attachment associated with and without bar connection in implant assisted mandibular overdenture (in vitro study)

**DOI:** 10.1186/s12903-023-03702-8

**Published:** 2023-12-08

**Authors:** Norhan M. Ameen, Nabila M. El-Khodary, Ahmed M. Abdel-Hamid, Aml E. Fahmy

**Affiliations:** 1 Department of implant and prosthodontics, Faculty of Dentistry, Pharos University, Alexandria, Egypt; 2 Faculty of Dentistry, Alexandria University, Alexandria, Egypt

**Keywords:** Implant-assisted overdenture, Low profile attachment, OT-Equator, OT-Equator bar, Strain gauges

## Abstract

**Background:**

The aim of this study was to compare the microstrain transmitted to peri-implant tissues of implant-assisted mandibular overdentures using two different low-profile attachment designs; OT- Equator attachment with and without bar attachment.

**Materials and methods:**

A completely edentulous epoxy resin mandibular model was used, in which two parallel dental implants were inserted at the canine region bilaterally and one in the middle. Sixteen identical complete edentulous mandibular overdentures were fabricated following conventional, standardized techniques and were divided equally between two groups according to the design and placement of the OT-Equator. Group A implants were kept solitary with an OT-Equator attachment, while group B implants were kept splinted with a bar associated with two mini-OT-Equator attachments in between. Sixteen identical mandibular complete overdentures were constructed, to which attachments were picked up. The difference in stress distribution was measured using strain gauges and compared between the two studied groups. A vertical load of 100 N using the universal testing machine was applied unilaterally on the left mesial fossae of the mandibular first molar and bilaterally on the bar attached to the mandibular premolar molar region of the overdentures. Statistical analysis was conducted using IBM SPSS version 28. Normality was checked by using the Shapiro-Wilk test and normality plots. The Mann-Whitney U test was then used to analogize the groups.

**Results:**

There was a statistically significant difference between groups A and B upon application of vertical unilateral and bilateral loadings of 100 N, with mean microstrain values of P 0.05. Group A (OT-Equator attachment) showed lower strain values than Group B (OT-Equator bar attachment) upon application of vertical, unilateral, and bilateral loadings of 100 N.

**Conclusions:**

Implant-assisted mandibular overdenture with a solitary attachment is associated with lower microstrain values around the implants after application of unilateral and bilateral vertical loadings of 100 N.

## Introduction

A complete denture is the standard treatment for a mandibular patient who is completely edentulous. Edentulous patients can have substantial difficulties using their conventional complete dentures due to a lack of retention, support, and stability and the related compromise in chewing ability. Endosseous implants have been shown to be a valuable rehabilitation for completely edentulous patients. A mandibular implant overdenture(OD) has been shown to improve masticatory function and patient satisfaction in complete denture patients who prefer an implant overdenture option [1].

Many different attachments available may be used to support implant-retained 64 overdenture (IRO). Mandibular IRO commonly uses ball attachments, a clip-on bar connecting the implants, or magnetic attachments [2]. Mandibular ODs are attached to implants using a wide range of commercially available attachment systems, either by splinting or unsplinting the implants [3].

The unique design and exceptionally low profile of the OT-Equator 4 in 1 System provides exceptional stability and superior retention when compared with other attachment systems. Due to its lower radius, it is indicated to correct divergence up to 28 degrees between implants without affecting the functionality of the elastic nylon cap, which is available in a wide variety of retention levels [4].

The bar attachment consists of a metallic bar that braces at least two or more implants, crossing the edentulous ridge between them, and a sleeve (superstructure) consolidated in the overdenture that cuts over the first bar to hold the denture. The bar attachments are accessible in a wide assortment of structures; they could be prefabricated or custom-made [5].

Types of bar attachment are bar joints that allow some degree of movement around the bar during mastication, it enhances retention, support, stability, and splinting of the abutments and bar unit that allow rigid fixation for the OD. It has parallel walls that prevent rotation or vertical movement of the prosthesis [6].

The disadvantages of bar attachment are the bulkiness of the bar attachment, the requirement of more plaque removal skills, the need for well retentive retainers, technical skills, complicated rebasing and repair, the relative parallism of the abutments and plaque accumulation under the bar, and the excessive restorative space required [7].

The use of the OT Equator associated with the Elastic Seeger seems to be a suitable treatment to obtain good passivation of the bar when an implant-supported overdenture is made. In fact, this device compensates for the space between the bar and the retentive equator of the abutment, creating a solid system. The tolerance between the bar and the OT Equator abutment has been designed to compensate for any misalignments that can be produced during the impression and the pouring of the cast model [8].

## Materials and methods

This study was conducted to compare the transmission of microstrain to peri-implant tissues of OT-Equator attachments with Smart Box housings associated with and without bar attachment in implant-assisted mandibular ODs after application of two different vertical loading techniques.

The sample size was calculated by the Mann-Whitney test using G-Power software. The total sample size was determined to be 16 samples that were grouped into 2 groups based on the design of the attachment.

Group A consists of three solitary OT-Equator attachment housings (SOT) for edentulous mandibular ODs (N = 8). While group B consists of edentulous mandibular ODs with two mini-OT-Equator attachments (BWO) and a space for a bar (N = 8).

### Experimental models and guide fabrication

A model was constructed from epoxy resin (Ramses Medical Products Factory) using a cast former as a replica of an average-sized edentulous mandible with an intercuspidal width of 29 mm, with the vibrator machine working at maximum speed to prevent air bubble formation in the final cast. A uniform 2-mm thick layer of a flexible polyurethane mucosa-mimicking material (Mollosil, Detax Co.) was applied to the model to replicate the ridge mucosa, and excess material was removed with a sharp instrument [9].

Fabrication of the maxillary and mandibular trial denture bases with wax occlusal rims were constructed on the duplicated stone model and mounted on a mean value articulator. The same set of mandibular acrylic teeth (size 22) (Acrostone, Co Ltd) were arranged on the trial denture bases.

**Fig. 1:**
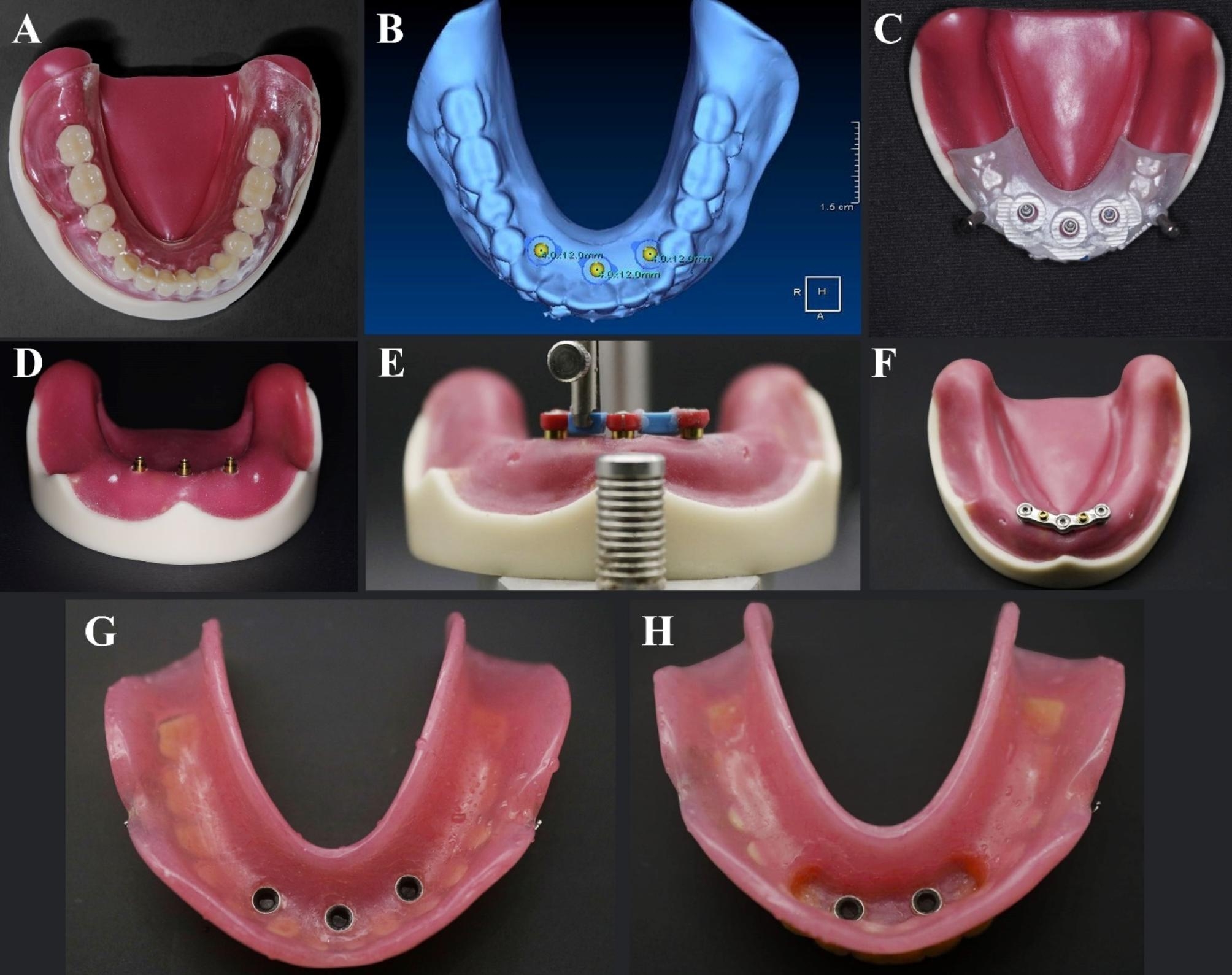
**A**. Conventional complete denture on epoxy model, **B**. Implant positioning plane, **C**. implant positioned, **D**. OT-Equator attachments placed onto the implants, **E**. Mount the bar components using mandrel, **F**. Finished metal bar, **G**. Pick up of three retentive caps in tissue surface of overdenture, **H**. Pick up of two retentive caps in tissue surface of overdenture

For the mandibular trial denture, flasking and packing were carried out using heat-curable polymethyl methacrylate (Acrostone Co Ltd) (Figure 1 A). The denture was prepared for scanning by making notches on the polished surface with a round bur. Gutta percha markers were placed in the mandibular denture.

The dual scan procedure by CBCT (Vatech) was used to fabricate a partially guiding CAD/CAM surgical guide (Clear Guide Data Shee, EnvisionTec,). Three B&B implants (Length: 12 mm and width: 4 mm) were virtually placed in almost the middle and canine regions using OnDemand software, considering the bone dimensions and anatomical limitations (Figure 1B).

The surgical guide was designed, constructed, and then fixed on the epoxy model with fixation pins. Using a rotary tissue punch, tags from the mucosal simulating material were removed. A universal surgical kit (In2guide universal kit – Cybermed) was used to drill into the epoxy model using the recommendations of the manufacturer.

B&B dummy implants were partially placed in the model through the guide; insertion of three implants of 12 mm length and 4 mm diameter were carried out using a torque wrench, and the primary stability was 35 Ncm (Figure 1 C). The top margins of the implant body are adjusted 1 mm below the level of the ridge crest.

### Bar fabrication

The same model was used for the two groups. According to the design of attachment used, in group A (the control group), implants were kept solitary with an OT-Equator, whereas in group B (the study group), implants were splinted with a bar connected to two mini-OT-Equator attachments in between three implants.

In group A (SOT) (the control group), three OT-Equator abutments (Rhein83) were screwed to each implant using a torque wrench torque of 20 Ncm and checking the OT-Equators’ parallelism using a dental surveyor (Figure 1D). While at group B (BWO) (the study group), three elastic seegers with cylinders (Rhein83) were applied to the OT-Equator abutments for passive bar connections system. A wax bar splinting the three red cylinders with elastic Seegers was fabricated using an OT bar multiuse spare parts permit a 2 mm space below the bar for hygienic reasons. The wax bar was fixed to the seegers using duralay material (Figure 1E).

Once the bar has been connected, create an area where the upper two mini-OT-Equators bar attachments will be positioned and combined with the bar. Vaseline was applied to the base of the attachment spacer as a separating material, and the attachment analogue was put on using the parallelometer key of the dental surveyor. The bar wax was finished, sprued, and casted as in the conventional, standardised wax-loss technique.

The metal bar was finished and returned to the model. Place the mini-OT-Equators on top of the bar using the parallelometer key and bond it to the sleeve with an OT-CEM self and photo-curing metal-to-metal bonding resin (Rhein83) in the cylinder. (Figure 1F).

### Fabrication of the mandibular overdentures

After model preparation, a polyvinyl siloxane impression was made using a stock tray and polyvinyl siloxane impression material to create room for further metal housing pick-up. This resulted in two duplicates of the model: one with three solitary OT-Equators (group A) and the other with a bar with two mini-OT-Equators on top (group B).

On the mounted duplicate stone models, sixteen identical mandibular trial denture bases were constructed. For each group, eight mandibular trial denture bases were constructed. In order to create sixteen identical trial dentures, the same set of mandibular acrylic teeth (size 22) were arranged on the record blocks while the opposite maxillary trial dentures were held in place using silicone indexes.

For the mandibular trial ODs, flasking and packing with heat-cure polymethyl methacrylate (Acrostone, Co Ltd) were carried out. The ODs were finished and polished using the traditional technique. (Figure 1G, Figure 1H)

### Pick-up of the attachments metal housing

Three OT-Equators were attached to the opposite implant using a torque wrench at a 20 Ncm torque. A metallic housing was then positioned over each abutment and pressed down to make sure the abutment engaged after a white block-out spacer ring was placed around each abutment.

The Group A ODs were placed to ensure that the caps were in full contact with the acrylic OD. To observe the full seating and to let out excess material, lingual vent openings were created all over the ODs.

Self-cure PMMA had been mixed and applied in the OD relief areas, then sat over the caps and stayed until the material was cured. Finishing and polishing were done on acrylic bases. For the Group B ODs using the same methods the bar was then screwed over the OT-Equator abutments and picked up the housing covers.

The black processing male component was removed and substituted with a clear OT-Equator retentive cap (standard elastic retentive cap) by using the retention insert tool (Zest Anchors, Inc.). The OD was then seated and pressed down to engage the elastic caps on the abutments.

At least 4 mm of the silicone mucosal simulation was removed from the mesial, distal, labial, and lingual areas around the implants to permit bonding of strain gauges to the epoxy resin model. The epoxy resin surfaces were flattened with a fissure bur, as recommended by the manufacturer [10, 11]. The surfaces were then smoothened using a fine grit sandpaper to obtain a surface texture suitable for strain gauge bonding and to avoid apparent incremental strain. ten linear strain gages (Type KFG-1- 120-C1-11L1M2R; gage length, 1 mm; gage resistance, 119.6 ± 0.4 Ω; adaptable thermal expansion, 11.7 PPM/C; temperature coefficient of gage factor, + 10,118%/C; gage factor, 2.08 ± 1.0%) were bonded to the epoxy resin at the mesial (M), distal (D), labial (LA), and lingual (L) surfaces of each implant.

A strain gauge adhesive (Kyowa Electronic Instrument Co., Ltd.) was used to monitor the strain around the implants during load application. The gauges were oriented mesiodistally perpendicular and labiallolingually parallel to the implant axes. A static load of 100 N was applied to the occlusal surface at the premolar molar areas using a loading device to calibrate the gauges.

**Fig. 2:**
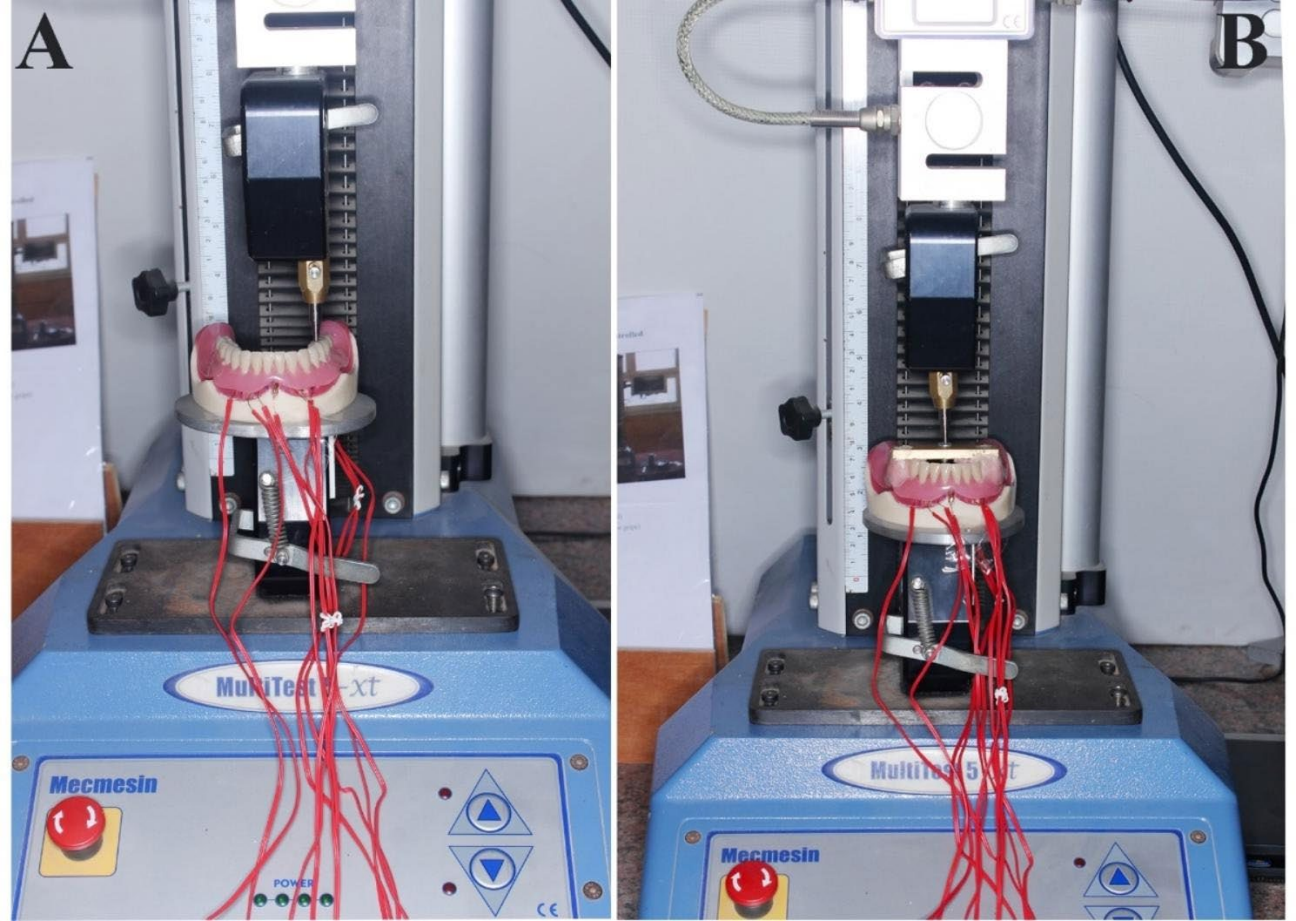
**A**. Unilateral load application, **B**. Bilateral load application

A universal testing machine (UTM) (Model-2006, Instron Corp.) [12] was used to deliver vertical static loads of 100 N (Figure 2 A) unilaterally (to simulate chewing on the preferred side) and bilaterally (Figure 2B) (to simulate clenching in centric occlusion) [13]. This amount of force represents a moderate level of biting force on IRO. The load was applied in compression mode at a constant rate (cross-head speed) of 0.5 mm/min.

For unilateral load application, the load was applied by the tip of the metal rod in the mesial fossae of the first molar of the mandibular OD; the loading was on the left side while the non-loading was on the right side. While applying bilateral load to the OD, the metal bars were constructed and cemented in the premolar molar areas of the OD with acrylic resin.

The tip of the metal rod was applied into the center of the bar in a compression mode, which was connected between the right and left premolar molar areas to deliver the bilateral load application. Electric signals from the four strain gauges were collected at a rate of 2 Hz (two readings per second) and amplified, transmitted, and recorded using a software package (Kyowa PCD, Kyowa Electronic Instruments Co.). All experiments were repeated five times for each OD, and the mean recorded microstrain was subjected to statistical analysis.

The strain gauge meter was calibrated before each measurement, The aim of calibration is to determine the relationship between the load applied and the strain signals received from the strain meter. The strain meter was adjusted to confirm the gauge factor of the strain gauges used. This adjustment was checked at regular intervals. The calibration process aimed to verify the linear association between the applied load and the resultant strain and assess the repeatability of the measurements.

Data were checked for normality using the Shapiro-Wilk test and normality plots, and it showed a non-normal distribution. Therefore, it was presented mainly by the median and inter quartile range in addition to the mean and standard deviation. Nonparametric tests were performed. Comparison between groups was done using the Mann-Whitney U test, while comparison between implants and surfaces within the same groups was done using the Friedman test and followed by a post hoc test with Bonferroni correction.

## Results

**Graph 1:**
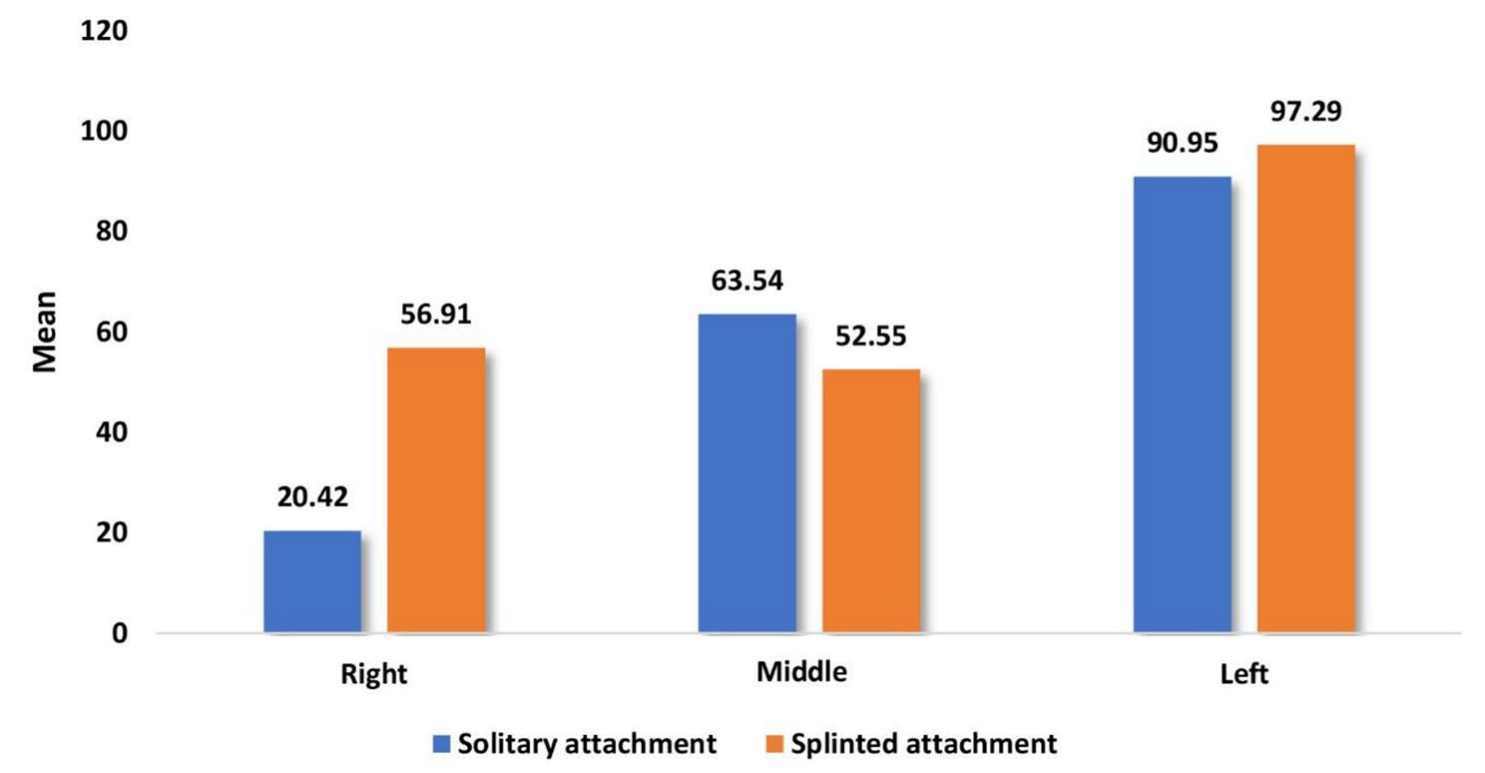
Comparison of overall mean and median of microstrain values between overdentures with solitary and splinted attachments during unilateral vertical load application for different implant positions

Table 1 and Graph 1 showed the overall mean and median of microstrains on all surfaces at the three implant locations. When unilateral loading of 100 N was applied, the OD with SOT attachments showed statistically lesser microstrains than the OD with BWO attachment at all three implant locations. In addition, the left implant at the side of the associated unilateral loading was statistically greater microstrains.

**Table 1:** Comparison of overall mean and median of microstrain values between overdentures with solitary and splinted attachments during unilateral vertical load application for different implant positions

Implant surface	Implant position	Solitary attachment (n = 8)		Splinted attachment (n = 8)		*P* value
		Mean (SD)	Median (IQR)	Mean (SD)	Median (IQR)	
Implants	Right	20.42 (0.89)	20.33 (1.38)	56.91 (2.74)	57.96 (5.00)	0.001*
	Middle	63.54 (1.08)	63.48 (1.71)	52.55 (2.38)	53.36 (4.45)	0.001*
	Left	90.95 (2.85)	89.25 (5.51)	97.29 (2.80)	97.31 (4.43)	0.002*
	***P*** **value**	< 0.0001* *P*_1_ = 0.137, *P*_2_ < 0.0001*, *P*_3_ = 0.137		0.001* *P*_1_ = 0.401, *P*_2_ = 0.073, *P*_3_ = 0.001*		

*Statistically significant differences at *P* value ≤ 0.05, *P*_1_: comparison between right implant and midline implant, *P*_2_: comparison between right implant and left implant, *P*_3_: comparison between midline implant and left implant

In addition, when comparing the macrostrains around the three implants at each surface, both ODs with two different attachment designs were associated with statistically greater microstrain values at the left implant at all their surfaces (side of loading).

**Graph 2:**
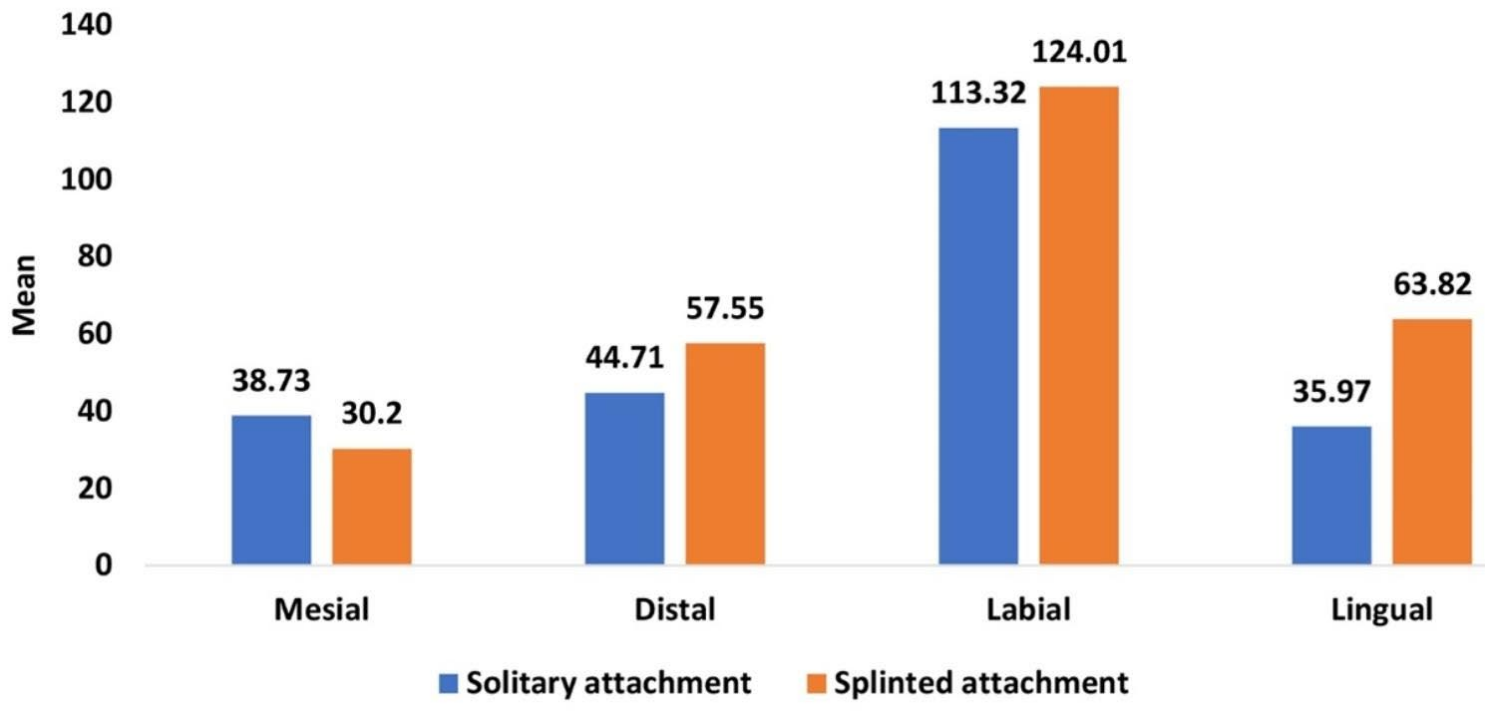
Comparison of overall mean and median of microstrain values during unilateral vertical load application between overdentures with solitary and splinted attachments for different implant surfaces

Table 2 and Graph 2 showed the overall mean and median of microstrain values of different surfaces (distal, mesial, labial, and lingual) around implants (right, middle, and left) and the comparison between them. When applying unilateral loading of 100 N on the left side (loaded side) of ODs, statistically greater microstrain values were recorded at the labial surface of both ODs with two different attachment designs in comparison with the other surfaces.

**Table 2:** Comparison of overall mean and median of microstrain values during unilateral vertical load application between overdentures with solitary and splinted attachments for different implant surfaces

Implant surface	Implant position	Solitary attachment (n = 8)		Splinted attachment (n = 8)		*P* value
		Mean (SD)	Median (IQR)	Mean (SD)	Median (IQR)	
Surfaces	Mesial	38.73 (1.20)	39.17 (2.17)	30.20 (2.17)	30.28 (3.59)	0.001*
	Distal	44.71 (3.37)	43.09 (6.91)	57.55 (4.18)	57.44 (8.20)	0.001*
	Labial	113.32 (2.08)	113.49 (3.98)	124.01 (2.51)	124.19 (5.12)	0.001*
	Lingual	35.97 (3.46)	33.70 (6.75)	63.82 (3.27)	64.79 (6.24)	0.001*
	***P*** **value**	< 0.0001* *P*_1_ = 0.199, *P*_2_ = 0.001*, *P*_3_ = 1.00, *P*_4_ = 0.728, *P*_5_ = 0.071, *P*_6_ < 0.0001*		< 0.0001* *P*_1_ = 0.317, *P*_2_ < 0.0001*, *P*_3_ = 0.040*, *P*_4_ = 0.040*, *P*_5_ = 1.00, *P*_6_ = 0.317		

*Statistically significant differences at *P* value ≤ 0.05, *P*_1_: comparison between mesial and distal, *P*_2_: comparison between mesial and buccal, *P*_3_: comparison between mesial and lingual, *P*_4_: comparison between distal and buccal, *P*_5_: comparison between distal and lingual, *P*_6_: comparison between buccal and lingual

**Graph 3:**
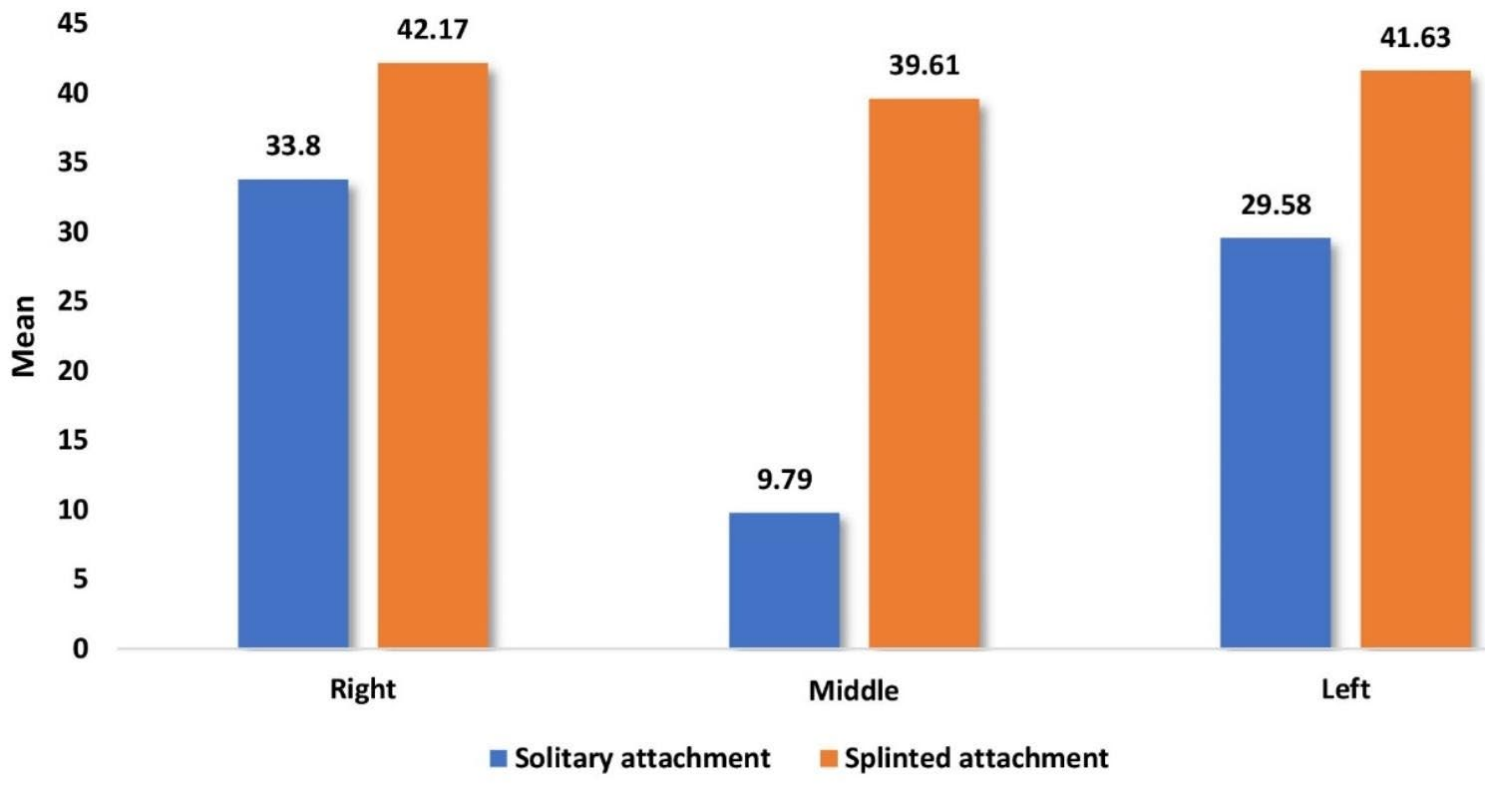
Comparison of overall mean and median of microstrain values between overdentures with solitary and splinted attachments during bilateral vertical load application for different implant positions

Table 3 and Graph 3 showed the overall mean and median of microstrains on all surfaces at the three implant locations. When bilateral loading of 100 N was applied, the OD with SOT attachment showed statistically lesser microstrains than the OD with BWO attachment at all three implant locations.

**Table 3:** Comparison of overall mean and median of microstrain values between overdentures with solitary and splinted attachments during bilateral vertical load application for different implant positions

Implant surface	Implant position	Solitary attachment (n = 8)		Splinted attachment (n = 8)		*P* value
		Mean (SD)	Median (IQR)	Mean (SD)	Median (IQR)	
Implants	Right	33.80 (1.06)	34.18 (1.40)	42.17 (3.86)	43.13 (6.93)	0.001*
	Middle	9.79 (0.53)	9.87 (0.54)	39.61 (2.09)	39.33 (3.39)	0.001*
	Left	29.58 (2.24)	29.48 (3.48)	41.63 (1.48)	41.92 (1.84)	0.001*
	***P*** **value**	< 0.0001* *P*_1_ < 0.0001*, *P*_2_ = 0.137, *P*_3_ = 0.137		0.197		

*Statistically significant differences at *P* value ≤ 0.05, *P*_1_: comparison between right implant and midline implant, *P*_2_: comparison between right implant and left implant, *P*_3_: comparison between midline implant and left implant

**Graph 4:**
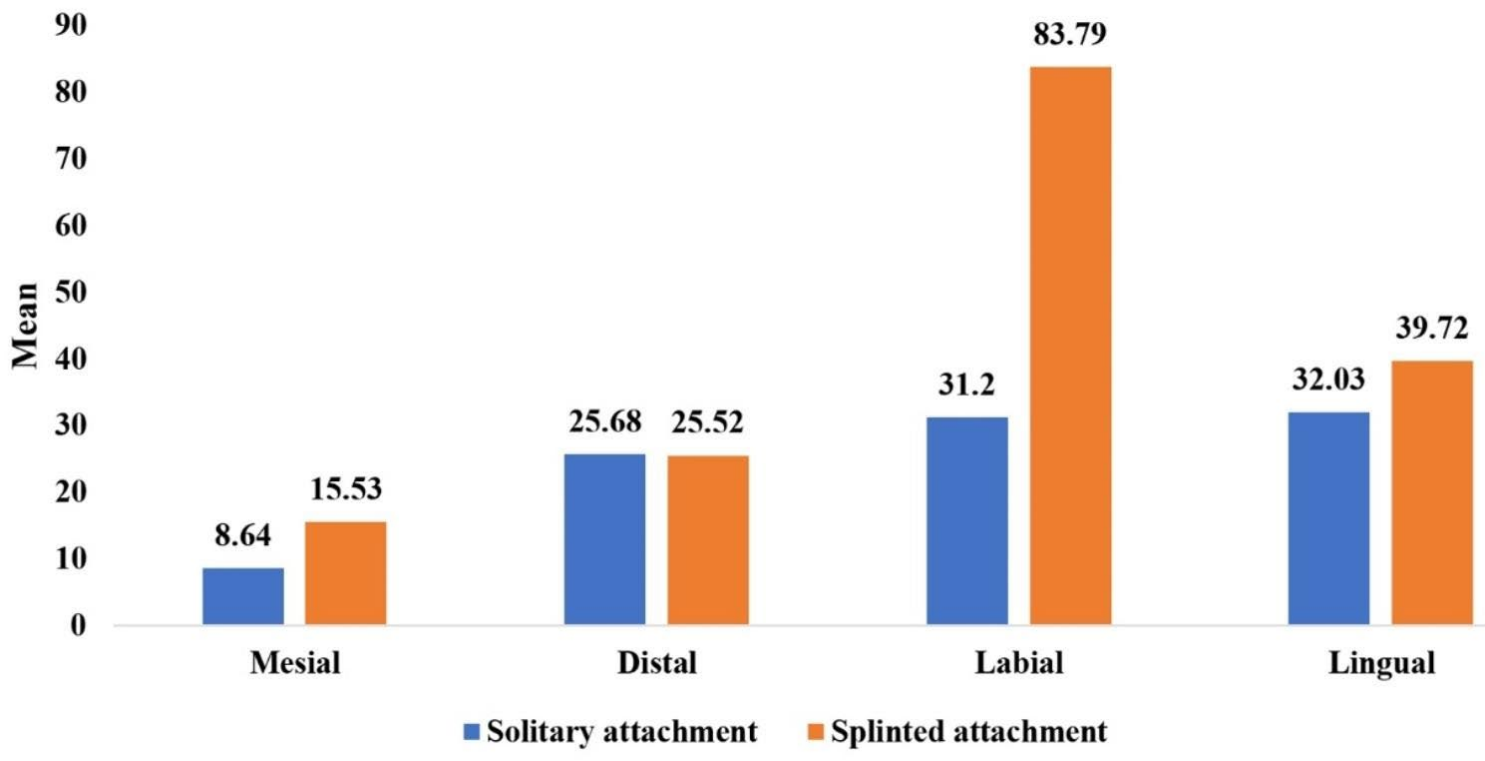
Comparison of overall mean and median of microstrain values during bilateral vertical load application between overdentures with solitary and splinted attachments for different implant surfaces

Table 4 and Graph 4 showed the overall mean and median of microstrain values of different surfaces (distal, mesial, labial, and lingual) around implants (right, middle, and left) and the comparison between them. When applying bilateral loading of 100 N on ODs, the statistically greater microstrain values were recorded at the labial and lingual surfaces of ODs with SOT attachment design, while the statistically greater microstrain values were recorded at the labial surface of ODs with BWO attachment design, in comparison with the other surfaces of different implant locations.

**Table 4:** Comparison of overall mean and median of microstrain values during bilateral vertical load application between overdentures with solitary and splinted attachments for different implant surfaces

Implant surface	Implant position	Solitary attachment (n = 8)		Splinted attachment (n = 8)		*P* value
		Mean (SD)	Median (IQR)	Mean (SD)	Median (IQR)	
Surfaces	Mesial	8.64 (1.08)	8.43 (1.91)	15.53 (1.82)	15.03 (3.83)	0.001*
	Distal	25.68 (2.03)	26.07 (3.61)	25.52 (2.50)	26.07 (4.76)	0.959
	Labial	31.20 (2.73)	29.96 (4.65)	83.79 (3.56)	82.43 (5.47)	0.001*
	Lingual	32.03 (1.28)	32.26 (2.23)	39.72 (2.70)	40.79 (5.12)	0.001*
	***P*** **value**	< 0.0001* *P*_1_ = 0.728, *P*_2_ = 0.003*, *P*_3_ < 0.0001*, *P*_4_ = 0.317, *P*_5_ = 0.040*, *P*_6_ = 1.00		< 0.0001* *P*_1_ = 0.728, *P*_2_ < 0.0001*, *P*_3_ = 0.012*, *P*_4_ = 0.012*, *P*_5_ = 0.728, *P*_6_ = 0.728		

*Statistically significant differences at *P* value ≤ 0.05, *P*_1_: comparison between mesial and distal, *P*_2_: comparison between mesial and buccal, *P*_3_: comparison between mesial and lingual, *P*_4_: comparison between distal and buccal, *P*_5_: comparison between distal and lingual, *P*_6_: comparison between buccal and lingual

**Graph 5:**
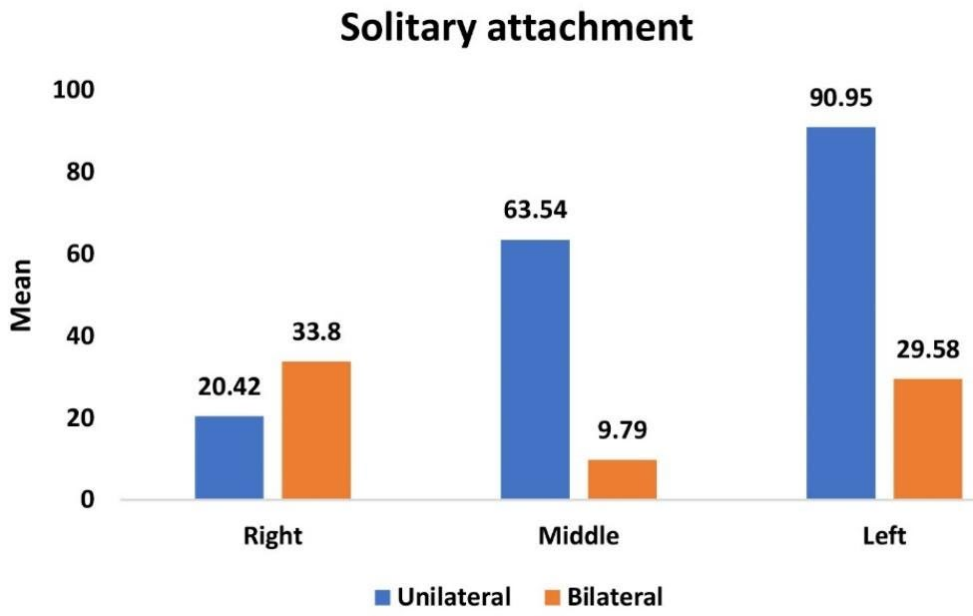
Comparison of overall mean and median of microstrain values of overdentures with solitary attachments upon application of unilateral and bilateral vertical load for different implant locations

Table 5 and Graph 5 showed the overall mean and median of microstrain values on all surfaces at the three implant locations upon application of unilateral and bilateral vertical of 100 N applications and the comparison between them. On SOT groups when applying the two different load techniques the application of bilateral load on ODs, statistically significant lesser microstrain values than the application of unilateral load on ODs.

**Table 5:** Comparison of overall mean and median of microstrain values of overdentures with solitary attachments upon application of unilateral and bilateral vertical load for different implant locations

Implant position	Unilateral load on ODs with Solitary (SOT) (n = 8)		Bilateral load on ODs with solitary (SOT) (n = 8)		*P* value
	Mean (SD)	Median (IQR)	Mean (SD)	Median (IQR)	
Right	20.42 (0.89)	20.33 (1.38)	33.80 (1.06)	34.18 (1.40)	< 0.0001*
Middle	63.54 (1.08)	63.48 (1.71)	9.79 (0.53)	9.87 (0.54)	< 0.0001*
Left	90.95 (2.85)	89.25 (5.51)	29.58 (2.24)	29.48 (3.48)	< 0.0001*

*Statistically significant differences at *P* value ≤ 0.05

**Graph 6:**
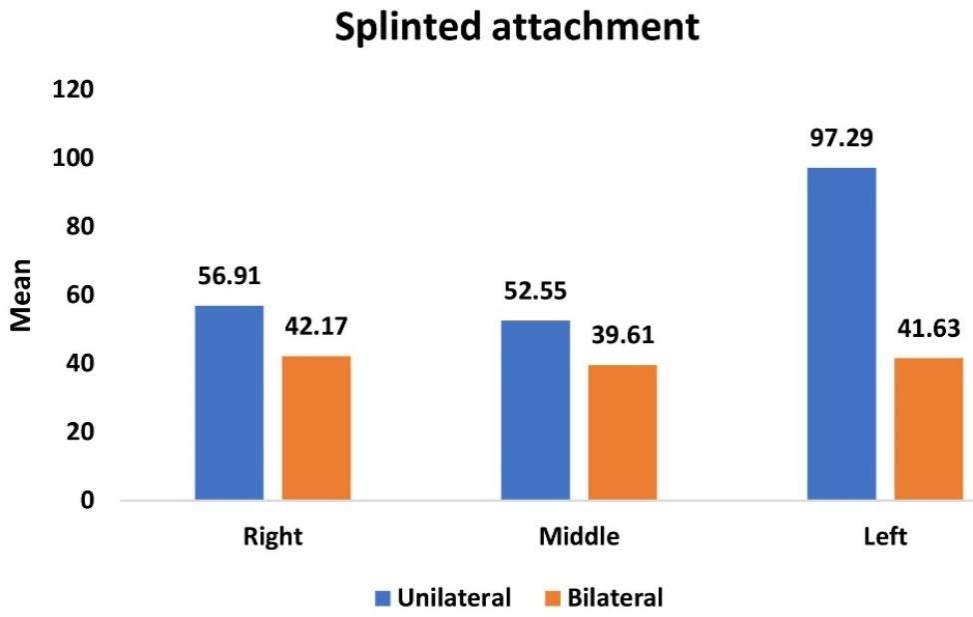
Comparison of overall mean and median of microstrain values of overdentures with splinted attachments upon application of unilateral and bilateral vertical load for different implant locations

Table 6 and Graph 6 showed the overall mean and median of microstrain values on all surfaces at the three implant locations upon application of unilateral and bilateral vertical of 100 N applications and the comparison between them. On BWO groups when applying the two different load techniques the application of bilateral load on ODs, statistically significant lesser microstrain values than the application of unilateral load on ODs.

**Table 6:** Comparison of overall mean and median of microstrain values of overdentures with splinted attachments upon application of unilateral and bilateral vertical load for different implant locations

Implant position	Unilateral load on ODs with Splinted (BWO) (n = 8)		Bilateral load on ODs with splinted (BWO) (n = 8)		*P* value
	Mean (SD)	Median (IQR)	Mean (SD)	Median (IQR)	
Right	56.91 (2.74)	57.96 (5.00)	42.17 (3.86)	43.13 (6.93)	< 0.0001*
Middle	52.55 (2.38)	53.36 (4.45)	39.61 (2.09)	39.33 (3.39)	< 0.0001*
Left	97.29 (2.80)	97.31 (4.43)	41.63 (1.48)	41.92 (1.84)	< 0.0001*

*Statistically significant differences at *P* value ≤ 0.05

## Discussion

The null hypothesis that the use of an OT-Equator attachment with or without a bar attachment would have no difference in influence on the stresses transmitted to the surrounding bone when used in an implant-assisted mandibular OD was rejected.

The results obtained from this study revealed that the microstrain values that were produced after unilateral and bilateral vertical load applications for the ODs with three-SOT attachments showed lesser microstrain values compared to ODs with the BWO attachment system at all implant positions and at all surfaces around each implant.

The load was distributed on three SOTs in the control group. While in the study group, the load was distributed on two mini-OT-Equators combined with the bar, resulting in a higher number of attachments, which is also thought to be beneficial in reducing stresses in the SOTs group.

The level of strain deformation with the OT-Equator attachment was smaller than that with the Equator bar attachment, and this can be explained by the fact that the vertical pressure was absorbed by the deformation of the components of the OT-Equator attachment and the ODs. This result is in agreement with Shin Takeshta et al. [14], who investigated the influence of different OD attachment systems on the stress generated in peri-implant bone during OD dislodgement and loading and found that during vertical dislodgement, the bar attachment showed the largest strain around the implant, followed by the ball attachment and the magnetic attachment.

Also in agreement with the study by Jin Suk Yoo et al. [13], who concluded that the level of strain deformation with the locator attachment was smaller than that with the bar/clip attachment, this can be explained by the fact that the vertical pressure was absorbed by the deformation of the components of the locator attachment and the denture.

The results of this study are contrary to those of Kivanç Akça et al. [15], who compared the biomechanical effect of splinted versus unsplinted mandibular implants supporting ODs subjected to an experimental static immediate load on bone tissue deformation using strain gauge analysis. According to the authors, the splinting of two interforaminal dental implants, regardless of attachment type, to support mandibular ODs subjected to immediate load significantly reduced the initial bone tissue strains experienced on the labial cortical bone in comparison with the use of unsplinted implants.

On the contrary, Leão et al. [16] conducted a meta-analysis comparing splinted and unsplinted overdenture attachment systems and found similar results between splinted and unsplinted OD attachment systems regarding marginal bone loss, implant survival rate, and prosthetic complications.

The results of this study have also shown lower microstrain values in the three-SOT attachment system under bilateral vertical load application in comparison to the three-SOT attachment system under unilateral vertical load application around the implant.

Also, the results of this study have shown lower microstrain values in the BWO attachment system under bilateral vertical load application in comparison to the BWO attachment system under unilateral vertical load application around the implant.

The left-side implant closer to the unilateral side (side of loading) was associated with greater microstrain values than the middle and right implants. Also, the labial surface of all implant positions was associated with greater microstrain values. This may be attributed to the anterior rotation of ODs between the fulcrum of rotation between the bilateral distal implants.

This result is in agreement with the results of Angel A. Arenal et al. [17], who evaluated and compared the stress distribution in locator attachments in mandibular two-implant ODs according to implant locations and different loading conditions using finite element analysis. According to the authors, the bilateral load in general favours a more uniform stress distribution in both attachments compared to the much greater stress registered with the unilateral load in the load-side attachments.

This result is in agreement with the results of Mohammed A. Mousa et al. [18] that the removable complete prostheses were subject to different kinds of stress in the form of compressive and tensile strengths. The buccal flanges exhibit compressive strains responding to the vertical forces, while the labial flanges show the same type of strain responding to all force directions. The highest tensile strain concentration exists at the anterior frenum, midline, and buccal flanges when the forces are horizontal.

There were a few drawbacks to this study, the mono-directional forces were used in an invitro test that would not reflect a practical model for a patient’s condition with OD. As well as a lack of in vivo simulations of salivary involvement, force of mastication, and muscle strength.

Further in vitro studies are recommended to evaluate retention behavior following the usage of the OT-Equator bar attachment compared to other splinted attachment designs, and the use of overdentures with solitary OT-Equator attachments in implant-assisted overdentures is successfully recommended, as it has proven to be a favourable attachment regarding stress distribution.

## Conclusions

The overdentures with a solitary OT-Equator attachment had lesser microstrain values at the three implant locations and different surfaces upon application of unilateral and bilateral 100 N vertical loading.

Unilateral vertical load showed higher microstrain values compared to bilateral vertical load among overdentures with both attachment designs. The lowest peri-implant strain was recorded around the solitary OT-Equator, especially during bilateral load application.

For overdentures with both attachment designs, the microstrain values were greater at the labial surface of different implant locations.

## Data Availability

All datasets and materials used and/or analyzed during the current study are included in this published article.

## Declarations

I hereby declare that no part of this dissertation has been submitted to Pharos University or to any other university in Egypt or abroad as part of the requirements to obtain another degree. Furthermore, Pharos University code of ethics and rules of academic integrity have been followed. Name: Norhan Moustafa Ameen.

## Abbreviations

IRO: Implant Retained Overdenture
OD: Overdenture
CAD: Computer Aided Design
CAM: Computer Aided manufacturing
BWO: Bar With OT-Equator
SOT: Solitary OT-Equator
UTM: Universal Testing Machine
N: Newton
MM: millimeter
